# Evaluation on the use of Nanopore sequencing for direct characterization of coronaviruses from respiratory specimens, and a study on emerging missense mutations in partial *RdRP* gene of SARS-CoV-2

**DOI:** 10.1186/s12985-020-01454-3

**Published:** 2020-11-23

**Authors:** Wai Sing Chan, Chun Hang Au, Ho Yin Lam, Candy Ling Na Wang, Dona Ngar-Yin Ho, Yuk Man Lam, Daniel Ka Wing Chu, Leo Lit Man Poon, Tsun Leung Chan, Jonpaul Sze-Tsing Zee, Edmond Shiu Kwan Ma, Bone Siu Fai Tang

**Affiliations:** 1 Department of Pathology, Hong Kong Sanatorium and Hospital, Hong Kong, China; 2 School of Public Health, Li Ka Shing, Faculty of Medicine, The University of Hong Kong, Hong Kong, China

**Keywords:** Coronavirus, COVID-19, Flongle, MinION, Missense mutation, Nanopore, RdRP, SARS-CoV-2

## Abstract

Coronavirus disease 2019 (COVID-19) pandemic has been a catastrophic burden to global healthcare systems. The fast spread of the etiologic agent, severe acute respiratory syndrome coronavirus 2 (SARS-CoV-2), highlights the need to identify unknown coronaviruses rapidly for prompt clinical and public health decision making. Moreover, owing to the high mutation rate of RNA viruses, periodic surveillance on emerging variants of key virus components is essential for evaluating the efficacy of antiviral drugs, diagnostic assays and vaccines. These 2 knowledge gaps formed the basis of this study. In the first place, we evaluated the feasibility of characterizing coronaviruses directly from respiratory specimens. We amplified partial *RdRP* gene, a stable genetic marker of coronaviruses, from a collection of 57 clinical specimens positive for SARS-CoV-2 or other human coronaviruses, and sequenced the amplicons with Nanopore Flongle and MinION, the fastest and the most scalable massively-parallel sequencing platforms to-date. Partial *RdRP* sequences were successfully amplified and sequenced from 82.46% (47/57) of specimens, ranging from 75 to 100% by virus type, with consensus accuracy of 100% compared with Sanger sequences available (n = 40). In the second part, we further compared 19 SARS-CoV-2 *RdRP* sequences collected from the first to third waves of COVID-19 outbreak in Hong Kong with 22,173 genomes from GISAID EpiCoV™ database. No single nucleotide variants (SNVs) were found in our sequences, and 125 SNVs were observed from global data, with 56.8% being low-frequency (n = 1–47) missense mutations affecting the rear part of RNA polymerase. Among the 9 SNVs found on 4 conserved domains, the frequency of 15438G > T was highest (n = 34) and was predominantly found in Europe. Our data provided a glimpse into the sequence diversity of a primary antiviral drug and diagnostic target. Further studies are warranted to investigate the significance of these mutations.

## Background

At the time of writing, coronavirus disease 2019 (COVID-19) has affected 216 countries, areas or territories, with 9,843,073 confirmed cases and 495,760 confirmed deaths in 6 months from the outbreak in Wuhan, China [1]. In Hong Kong, the first 2 COVID-19 cases were confirmed on 23 January 2020 [2]. At that time, a number of severe acute respiratory syndrome coronavirus 2 (SARS-CoV-2) genome sequences and real-time reverse transcription polymerase chain reaction (rRT-PCR) protocols were already available so that we were more prepared than Wuhan for tracing and controlling circulation of this virus. Nevertheless, we cannot predict when and where the next coronavirus spillover will take place. Perhaps what we can do is to be well prepared based on accumulating knowledge on this virus family and well utilize state-of-the-art tools to facilitate early identification and timely containment. On the other hand, owing to the high mutation rate of RNA viruses, periodic surveillance on emerging variants of key virus components is essential to combat the viruses. Through studying their functional characteristics and evolution pattern, we can monitor and evaluate the impact of emerging variants on the efficacy of antiviral drugs, diagnostic assays and vaccines.

To control the spread of a highly contagious, unknown virus, rapid and accurate characterization of virus genome is crucial for developing sensitive screening assays. Metagenomic sequencing is a useful tool for rapid reconstruction of virus genomes, as evident by discovery and characterization of SARS-CoV-2 [3–5]. Successful retrieval of a complete virus genome from complex clinical specimens requires very deep sequencing to compensate contamination by host and commensal reads, with sequencing data processed by high performance computers and analyzed by bioinformatics expertise. As these are luxurious for most clinical laboratories, identification and characterization of unknown viruses are usually confined to reference laboratories. As a result, there is a lapse between initial presentation of a patient/ patients infected by unknown coronavirus, clueless microbiological investigations in frontline laboratories, and finally referral to reference laboratories for etiologic investigation. The duration of this lapse may determine the controllability of an outbreak. Compared with metagenomic sequencing, characterization of partial virus genome involves simpler workflow which is more implementable as a part of etiologic investigation in frontline laboratories, providing hint for more timely follow-up actions. This pan-coronavirus approach was also adopted for initial investigation of Middle East respiratory syndrome (MERS) and COVID-19 outbreaks [4–6].

**Fig. 1:**
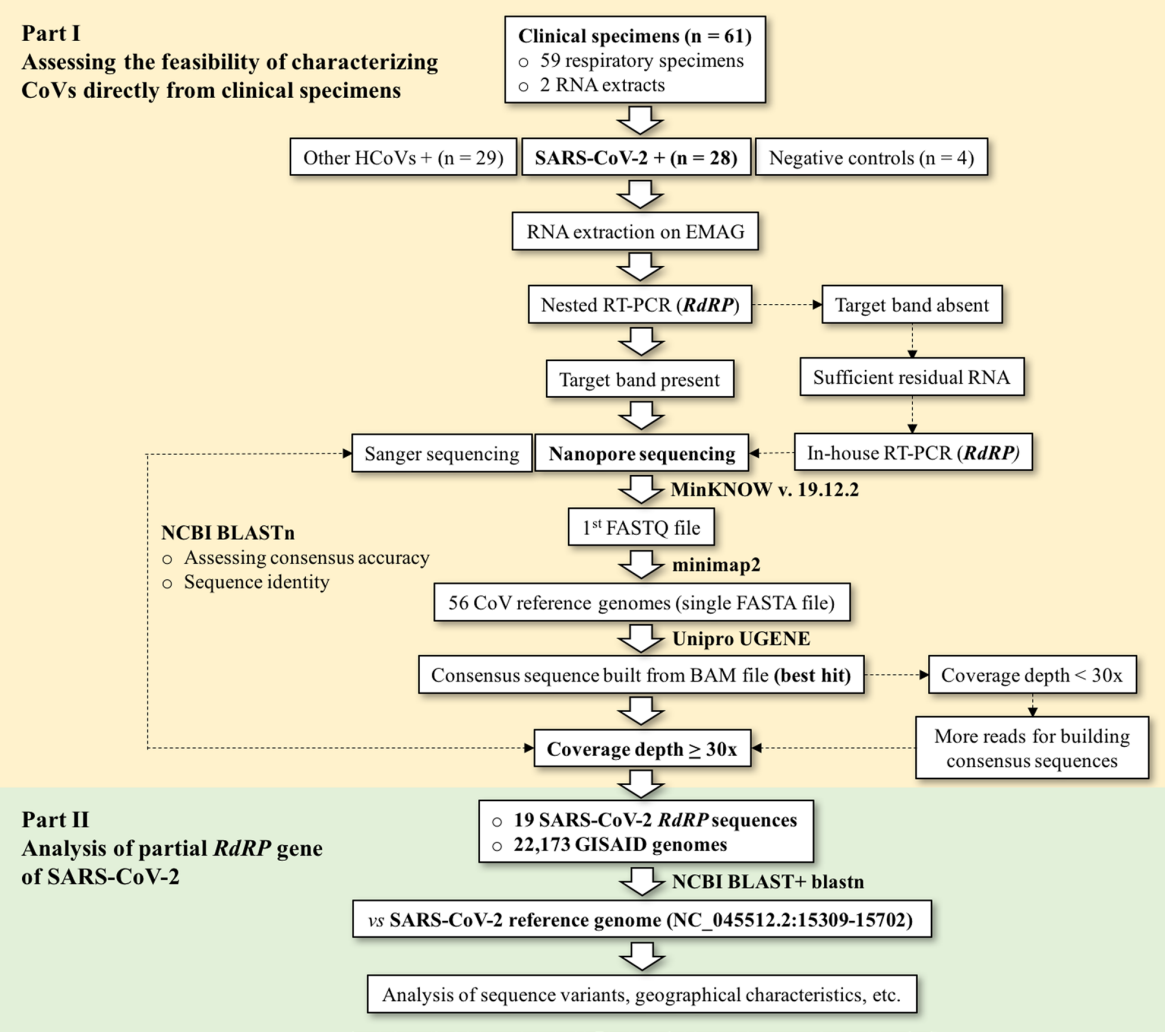
An overview of this study. The flowchart summarizes the workflow of this study. First, we assessed the feasibility of amplifying and sequencing partial *RdRP* gene directly from a collection of 61 clinical specimens. Second, we performed sequence analysis on 19 SARS-CoV-2 *RdRP* sequences from our study and 22,173 GISAID genomes, using SARS-CoV-2 reference genome Wuhan-Hu-1 (NC_045512.2:15309-15702) as reference

In the first part of this study, we evaluated the feasibility of characterizing coronaviruses directly from clinical specimens. We selected partial RNA-dependent RNA polymerase gene (*RdRP*) as the amplification target, as it has been commonly used for coronavirus classification and phylogenetic analysis [7, 8]. We sequenced the amplicons using Nanopore technology, which is the fastest and most scalable option in current massively-parallel sequencing market, and assessed its consensus accuracy with Sanger’s method. As COVID-19 pandemic is ongoing, every piece of genetic information about the causative agent may save lives. Therefore, in the second part of this study, we compared the SARS-CoV-2 *RdRP* sequences from our laboratory with genomes worldwide and looked for mutations which might alter the function of this key virus component. An overview of this study is shown in Fig. 1.

## Methods

### Specimens

A total of 61 clinical specimens were tested, among which 57 were positive for SARS-CoV-2, HCoV-229E, NL63, HKU1 or OC43 and 4 were negative controls (Table 1). The 2 RNA extracts of SARS-CoV-2 culture isolates were kindly provided by School of Public Health, Li Ka Shing Faculty of Medicine, The University of Hong Kong. Respiratory specimens were collected from 16 December 2019 to 16 August 2020 in Department of Pathology, Hong Kong Sanatorium & Hospital and routinely tested with rRT-PCR assays for SARS-CoV-2 [9] and/ or BIOFIRE® FILMARRAY® Respiratory 2 plus Panel (RP2plus, bioMérieux, Marcy I’Etoile, France).

**Table 1:** Results of routine RT-PCR assays and Nanopore sequencing

Specimen types		Routine RT-PCR test results		Nanopore sequencing results			
		HCoV	Ct value^¶^	Flow cell type	Run time	Consensus sequence length (No. of mapped reads)	Consensus accuracy (Sanger sequence length)
1^*^	VC	SARS-CoV-2	N: 29.65 orf1b: ND	Flongle	23 m	394 bp (268)	100% (394 bp)
2^*^	VC	SARS-CoV-2	N: 35 orf1b: ND	Flongle	32 m	394 bp (99)	100% (394 bp)
3^*^	NPS-TS	SARS-CoV-2	N: 29.8 orf1b: 30.42	Flongle	29 m	394 bp (248)	100% (297 bp)
4^*^	SP	SARS-CoV-2	N: 35 orf1b: 35	Sequencing was not performed due to absence of PCR target band			
5^#^	pOS	SARS-CoV-2	N: 28.8 orf1b: 28.66	Flongle	22 m	394 bp (895)	100% (394 bp)
6^#^	pOS	SARS-CoV-2	N: 28.1 orf1b: 28.41	Flongle	16 m	394 bp (932)	100% (394 bp)
7^#^	pOS	SARS-CoV-2	N: 32.96 orf1b: 33.94	MinION	3 h 18 m	394 bp (36)	100%^‡^
8^#^	pOS	SARS-CoV-2	N: 33.5 orf1b: 33.5	Sequencing was not performed due to absence of PCR target band			
9^#^	pOS	SARS-CoV-2	N: > 35 orf1b: > 35	Sequencing was not performed due to absence of PCR target band			
10^#^	pOS	SARS-CoV-2	N: > 35 orf1b: > 35	Sequencing was not performed due to absence of PCR target band			
11^#^	pOS	SARS-CoV-2	N: > 35 orf1b: > 35	MinION	Not detected		
12^†^	pOS	SARS-CoV-2	N: 14.5 orf1b: 15.48	MinION	17 m	394 bp (3420)	100% (394 bp)
13^†^	pOS	SARS-CoV-2	N: 15.6 orf1b: 16.67	MinION	31 m	394 bp (3036)	100% (394 bp)
14^†^	pOS	SARS-CoV-2	N: 19.2 orf1b: 19.92	MinION	31 m	394 bp (3449)	100% (394 bp)
15^†^	pOS	SARS-CoV-2	N: 19.52 orf1b: 21.2	MinION	22 m	394 bp (2176)	100% (394 bp)
16^†^	pOS	SARS-CoV-2	N: 21.1 orf1b: 21.76	MinION	23 m	394 bp (2617)	100% (394 bp)
17^†^	pOS	SARS-CoV-2	N: 22.1 orf1b: 23.27	MinION	17 m	394 bp (2484)	100% (394 bp)
18^†^	pOS	SARS-CoV-2	N: 22.6 orf1b: 22.77	MinION	7 m	394 bp (99)	100%^‡^
19^†^	pOS	SARS-CoV-2	N: 22.67 orf1b: 22.86	MinION	16 m	394 bp (3826)	100% (394 bp)
20^†^	pOS	SARS-CoV-2	N: 24.23 orf1b: 24.81	MinION	43 m	394 bp (58)	100%^‡^
21^†^	pOS	SARS-CoV-2	N: 24.5 orf1b: 25.19	MinION	31 m	394 bp (126)	100%^‡^
22^†^	pOS	SARS-CoV-2	N: 24.7 orf1b: 24.9	MinION	17 m	394 bp (2362)	100% (394 bp)
23^†^	pOS	SARS-CoV-2	N: 24.94 orf1b: 26.07	MinION	34 m	394 bp (54)	100%^‡^
24^†^	pOS	SARS-CoV-2	N: 27.76 orf1b: 28.9	MinION	1 h	394 bp (1)^§^	97.21%^‡§^
25^†^	pOS	SARS-CoV-2	N: 30.47 orf1b: 30.6	MinION	33 m	394 bp (1846)	100% (326 bp)
26^†^	pOS	SARS-CoV-2	N: 31.26 orf1b: 31.61	MinION	1 h	394 bp (2)^§^	99.24%^‡§^
27^†^	pOS	SARS-CoV-2	N: 31.68 orf1b: 32.23	MinION	Not detected		
28^†^	pOS	SARS-CoV-2	N: 35 orf1b: 35	MinION	Not detected		
29	NPS	HKU1	N/A	MinION	Not detected		
30	NPS	HKU1	N/A	MinION	52 m	394 bp (3804)	100% (394 bp)
31	NPS	HKU1	N/A	MinION	5 m	394 bp (3572)	100% (394 bp)
32	NPS	HKU1	N/A	MinION	6 m	394 bp (3877)	100% (394 bp)
33	NPA	229E	N/A	MinION	53 m	394 bp (4045)	100% (394 bp)
34	NPS	229E	N/A	MinION	51 m	394 bp (3908)	100% (394 bp)
35	NPS	229E	N/A	MinION	40 m	394 bp (4044)	100% (394 bp)
36	NPS	229E	N/A	MinION	1 h 27 m	394 bp (3882)	100% (394 bp)
37	NPS	229E	N/A	MinION	53 m	394 bp (4076)	100% (394 bp)
38	NPS	229E	N/A	MinION	46 m	394 bp (3971)	100% (394 bp)
39	NPS	229E	N/A	MinION	36 m	394 bp (4062)	100% (394 bp)
40	NPS	OC43	N/A	MinION	52 m	394 bp (3556)	100% (394 bp)
41	TS	OC43	N/A	Sequencing was not performed due to absence of PCR target band			
42	NPS	OC43	N/A	MinION	56 m	394 bp (4126)	100% (394 bp)
43	TS	OC43	N/A	MinION	1 h 8 m	394 bp (3977)	100% (394 bp)
44	NS	OC43	N/A	MinION	2 m	394 bp (3845)	100% (394 bp)
45	NS	OC43	N/A	MinION	46 m	394 bp (4120)	100% (394 bp)
46	NPS	OC43	N/A	MinION	1 m	394 bp (85)	100% (394 bp)
47	NPS	OC43	N/A	MinION	1 h 26 m	394 bp (1543)	100% (364 bp)
48	NPA	OC43	N/A	MinION	1 h 14 m	394 bp (4066)	100% (394 bp)
49	NPS	OC43	N/A	MinION	44 m	394 bp (3998)	100% (394 bp)
50	NPS	OC43	N/A	MinION	43 m	394 bp (4096)	100% (394 bp)
51	NPS	OC43	N/A	MinION	40 m	394 bp (4015)	100% (394 bp)
52	NPS	NL63	N/A	MinION	54 m	394 bp (4029)	100% (394 bp)
53	NPS	NL63	N/A	MinION	35 m	394 bp (4019)	100% (394 bp)
54	NPS	NL63	N/A	Flongle	47 m	394 bp (851)	100% (298 bp)
55	NPS	NL63	N/A	MinION	37 m	394 bp (4030)	100% (394 bp)
56	NPS	NL63	N/A	Sequencing was not performed due to absence of PCR target band			
57	NPS	NL63	N/A	MinION	1 h 11 m	394 bp (3898)	100% (394 bp)
58	SP	Not detected		MinION	Not detected		
59	SP	Not detected		MinION	Not detected		
60	NPS	Not detected		MinION	Not detected		
61	pOS	Not detected		MinION	Not detected		

^¶^N and orf1b correspond to nucleocapsid gene and open reading frame 1b, respectively. The results of BioFire® FilmArray® Respiratory 2 Panel were qualitative and therefore Ct values were not available

^*^Collected during the first wave of COVID-19 outbreak (January to early March, 2020, n = 4)

^#^Collected during the second wave of COVID-19 outbreak (mid-March to May, 2020, n = 7)

^†^Collected during the third wave of COVID-19 outbreak (late June onwards, 2020, n = 17)

^‡^Sanger sequence was not available due to high level of background noise. The Nanopore read/ consensus sequence was compared to SARS-CoV-2 reference genome (NC_045512.2)

^§^The number of Nanopore reads was insufficient for generating accurate consensus sequence (< 30×)

*Ct* threshold cycle, *HCoV* human coronavirus, *N/A* not available, *ND* not done, *NPA* nasopharyngeal aspirate, *NPS* nasopharyngeal swab, *NS* nasal swab, *pOS* posterior oropharyngeal saliva, *RT-PCR* reverse transcription polymerase chain reaction, *SP* sputum, *TS* throat swab, *VC* virus culture

### RNA extraction

Standard laboratory practices were applied to minimize risk of infection and contamination. RNA was extracted from 200–500 µL of respiratory specimens using EMAG® (bioMérieux, Marcy I’Etoile, France). Nasal swabs, nasopharyngeal swabs and throat swabs preserved in universal transport medium (UTM®, Copan, Murrieta, CA, USA) were homogenized by vortexing and added directly to NUCLISENS® lysis buffer (bioMérieux, Marcy I’Etoile, France). Posterior oropharyngeal saliva, nasopharyngeal aspirate and sputum were liquefied with equal volume of working sputasol (Oxoid, Poole, England), briefly centrifuged to sediment large cell debris, and 400 µL of supernatant was added to lysis buffer. Off-board lysis was performed at ambient temperature for 10 min before loading into EMAG® for total nucleic acid extraction, with elution volume of 50 µL. The extracts were kept on ice before testing or stored at −80 °C.

### Reverse transcription and pan-coronavirus PCR

Published primers [10, 11] were aligned to all known human coronavirus reference genomes (NC_002645.1, NC_006577.2, NC_005831.2, NC_006213.1, NC_004718.3, NC_019843.3 and NC_045512.2) to check for 3′ complementarity and adopted for nested amplification of partial *RdRP* gene (Table 2). QIAGEN OneStep RT-PCR Kit (Qiagen, Hilden, Germany) was used for reverse transcription and first PCR from 10 µL of RNA, followed by second PCR using AmpliTaq Gold™ DNA Polymerase (Applied Biosystems, Foster City, CA, USA). First PCR samples were purified using AMPure XP beads (Beckman-Coulter, Brea, CA, USA), and the 5-µL eluates were used for second PCR. Second PCR samples were electrophoresed on 2% agarose gel (Invitrogen, Carlsbad, CA, USA) and stained with 0.5 µg/mL ethidium bromide (Invitrogen, Carlsbad, CA, USA). Samples with visible band(s) around target size (440 bp) were sequenced directly by both Nanopore and Sanger’s methods.

**Table 2:** Published primers and nested RT-PCR conditions

Primer sequences		
*1st PCR*		
Forward	5′-GGN TGG GAY TAY CCN AAR TGY GA-3′	760 bp amplicon
Reverse	5′-RHG GRT ANG CRT CWA TDG C-3′	
*2nd PCR*		
Forward	5′-GGT TGG GAC TAT CCT AAG TGT GA-3′	440 bp amplicon
Reverse	5′-CCA TCA TCA GAT AGA ATC ATC AT-3′	

Reverse transcription & First PCR				
Master mix constituents per reaction		PCR profile		
5× RT buffer	5 µL			
dNTP (10 mM)	1 µL	45 °C	30 m	
5× Q solution	5 µL	95 °C	15 m	
Forward primer (200 µM)	0.5 µL	94 °C	30 s	
Reverse primer (200 µM)	0.5 µL	45 °C	30 s	50×
One-step enzyme mix	1 µL	72 °C	1 m	
RNase-free water	2 µL	72 °C	10 m	
RNA	10 µL	15 °C	Hold	
Total	25 µL			

Second PCR				
Master mix constituents per reaction		PCR profile		
10x PCR buffer	5 µL			
MgCl_2_ (25 mM)	3 µL	95 °C	10 m	
dNTP (10 mM)	1 µL	95 °C	15 s	
Forward primer (10 µM)	1 µL	45 °C	30 s	35×
Reverse primer (10 µM)	1 µL	72 °C	30 s	
PCR-grade water	33.5 µL	72 °C	5 m	
AmpliTaq Gold^™^	0.5 µL	15 °C	Hold	
Purified 1^st^ PCR amplicon	5 µL			
Total	50 µL			

For the 6 PCR-negative specimens with sufficient residual RNA (Specimen 18, 20, 21, 23, 26 and 27), RT-PCR was repeated using an in-house developed protocol (Table 3). A new set of primers were designed by aligning second PCR primers [11] to 56 coronavirus reference genomes, with degenerate bases added to appropriate positions. SuperScript® III First-Strand Synthesis System (Invitrogen, Carlsbad, CA, USA) was used for reverse transcription from 8 µL of RNA, followed by PCR using AmpliTaq Gold™ DNA Polymerase with 20 µL of cDNA. PCR was optimized with higher magnesium chloride concentration and slower ramp rate to allow better tolerance for variations at primer binding sites.

**Table 3:** In-house developed primers and RT-PCR protocol

Primer sequences		
Forward 1	5′-ATG GGN TGG GAY TAY CC-3′	~ 440 bp amplicon
Forward 2	5′-GGA YTA YCC NAA RTG YGA-3′	
Reverse	5′-CCA TCA TCA SWN ARN ATS AT-3′	

Reverse transcription			
Master mix 1 constituents per reaction		Temperature profile	
Random hexamer	1 µL	65 °C On ice	5 m 1 m
dNTP (10 mM)	1 µL		
RNA template	8 µL		
Total	10 µL		

Master mix 2 constituents per reaction		RT profile	
10x RT buffer	2 µL		
MgCl_2_ (25 mM)	4 µL	25 °C	10 m
DTT (0.1 M)	2 µL	50 °C	50 m
RNaseOUT	1 µL	85 °C	5 m
SuperScript III reverse transcriptase	1 µL	15 °C	Hold
Master mix 1	10 µL		
Total	20 µL		

PCR				
Master mix constituents per reaction		PCR profile		
10x PCR buffer	5 µL			
MgCl_2_ (25 mM)	8 µL			
dNTP (10 mM)	1 µL	95 °C	9 m	
Forward primer 1 (200 µM)	1 µL	95 °C (1 °C/s)	1 m	
Forward primer 2 (200 µM)	1 µL	48 °C (1 °C/s)	1 m	40×
Reverse primer (200 µM)	1 µL	72 °C (1 °C/s)	1 m	
PCR-grade water	11.5 µL	72 °C	5 m	
AmpliTaq Gold^™^	1.5 µL	15 °C	Hold	
RT product	20 µL			
Total	50 µL			

The primers were designed with reference to 56 coronavirus reference genomes

Coronavirus reference genomes considered for designing primers

NC_028752.1, NC_002645.1, NC_005831.2, NC_032107.1, NC_028824.1, NC_009988.1, NC_010437.1, NC_010438.1, NC_028814.1, NC_018871.1, NC_003436.1, NC_009657.1, NC_022103.1, NC_028811.1, NC_028833.1, NC_028806.1, NC_038861.1, NC_002306.3, NC_030292.1, NC_023760.1, NC_034972.1, NC_032730.1, NC_035191.1, NC_038294.1, NC_019843.3, NC_034440.1, NC_009020.1, NC_009019.1, NC_039207.1, NC_004718.3, NC_045512.2, NC_014470.1, NC_025217.1, NC_030886.1, NC_009021.1, NC_003045.1, NC_006213.1, NC_017083.1, NC_026011.1, NC_001846.1, AC_000192.1, NC_012936.1, NC_006577.2, NC_011547.1, NC_011549.1, NC_016993.1, NC_011550.1, NC_039208.1, NC_016992.1, NC_016991.1, NC_016996.1, NC_016994.1, NC_016995.1, NC_001451.1, NC_010800.1, NC_010646.1

### Sanger sequencing

Five microliters of PCR products were purified enzymatically using ExoSAP-IT™ (Affymetrix, Santa Clara, CA, USA), followed by cycle sequencing using BigDye™ Terminator v.1.1 Cycle Sequencing Kit (Applied Biosystems, Foster City, CA, USA). Sequencing products were purified using BigDye® XTerminator™ Purification Kit and analyzed on 3730 DNA Analyzer (Applied Biosystems, Foster City, CA, USA). Sanger consensus sequences were deprived of primers, and their identity and similarity to Nanopore consensus sequences were determined using NCBI BLASTn.

### Nanopore sequencing

Nanopore sequencing libraries were prepared using Ligation Sequencing Kit 1D and PCR-free Native Barcoding Expansion Kit (SQK-LSK109 and EXP-NBD104/114, Oxford Nanopore Technologies, Oxford, England) with 40 μL of amplicons as input. Sequencing and unique barcode adaptors were ligated to the reads following manufacturer’s recommendations. The libraries were loaded and sequenced on Flongle or MinION flow cells (FLO-FLG001 R9.4.1 or FLO-MIN106D R9.4.1, Oxford Nanopore Technologies, Oxford, England). Live basecalling and demultiplexing were facilitated by MinKNOW version 19.12.2.

### Bioinformatics

The bioinformatics workflow is summarized in Fig. 1. Nanopore sequencing reads from ‘fastq pass’ folders were used for data analysis. Reads from the first FASTQ files were aligned to 56 coronavirus reference genomes (downloaded from NCBI nucleotide database on 14 February 2020) using minimap2 (Galaxy version 2.17 + galaxy0) [12]. From the resulting BAM files, consensus sequences were built with best-matched reference using Unipro UGENE (version 1.29.0) and deprived of primers. If coverage depth was less than 30x, more sequencing reads would be used for consensus building to attain a minimum depth of 30x. Identity of consensus sequences and similarity to their Sanger counterparts were evaluated using NCBI BLASTn.

Full SARS-CoV-2 genomes were downloaded from Global Initiative on Sharing All Influenza Data (GISAID) EpiCoV™ database (accessed on 3 June 2020) with the following search criteria: collection date from 1 December 2019 to 31 May 2020, human host, complete genomes > 29,000 bp, and high coverage. Partial *RdRP* sequence was extracted from SARS-CoV-2 Wuhan-Hu-1 reference genome (NC_045512.2:15309-15702) and used as the reference for single nucleotide variant (SNV) analysis. The reference was aligned to partial *RdRP* sequences of SARS-CoV-2 from this study and GISAID EpiCoV™ using NCBI BLAST + blastn (Galaxy version 0.3.3) [13]. Sequences with alignment length of 394 bp and without unknown bases (N) were extracted for SNV analysis using Unipro UGENE (version 1.29.0). Distribution of missense mutations was studied from geographical (Africa, America, Asia/ Middle East, Europe and Oceania) and temporal (month of collection) perspectives. Number of sequences possessing a particular SNV was normalized by total number of genomes retrieved from that geographical area.

## Results

### Nanopore sequencing results

Results are shown in Table 1. Partial *RdRP* sequences were successfully amplified and sequenced from 82.46% (47/57) of positive specimens. Success rate by virus type was 75% (21/28) for SARS-CoV-2, 75% (3/4) for HCoV-HKU1, 100% (7/7) for HCoV-229E, 91.67% (11/12) for HCoV-OC43 and 83.33% (5/6) for HCoV-NL63. Among these 47 specimens, full-length consensus sequences were built from 45 specimens (95.74%), with minimum coverage depth of 30× and were identical to their Sanger counterparts, if available (n = 40). For Specimen 24 and 26, there were insufficient reads for building accurate consensus sequences, and their identities to SARS-CoV-2 reference genome were 97.21% and 99.24%, respectively. Nanopore run time ranged from 1 min to 3 h and 18 min.

### Consensus building without SARS-CoV-2 reference genome

To mimic characterizing an unknown coronavirus, we randomly selected 6 SARS-CoV-2-positive specimens (Specimen 1, 2, 3, 5, 6 and 7) and aligned their reads to all coronavirus reference genomes without SARS-CoV-2 Wuhan-Hu-1, and consensus sequences were built with best-matched reference. The best hit for Specimen 1, 2, 5, 6 and 7 was bat coronavirus BM48-31/BGR/2008 (NC_014470.1). The consensus sequences were 98.48% similar to those built with SARS-CoV-2 Wuhan-Hu-1, and their identity to bat coronavirus BM48-31/BGR/2008 was 89.82%. The discrepancy from original consensus sequences arose from 2 low-coverage bases (thymine and adenine, NC_014470.1:15484/15486) and 2 ‘insertions’ (cytosine and thymine, after bases 15485 and 15498, respectively) that were incorrectly excluded. For Specimen 3, the best hit was SARS coronavirus Tor2 (NC_004718.3). The consensus sequence was 99.24% similar to that built with SARS-CoV-2 Wuhan-Hu-1, and its identity to SARS coronavirus Tor2 was 88.78%. A low-coverage thymine (NC_004718.3:15584) and a ‘false insertion’ of cytosine (after base 15586) were found in alignment data.

### Partial *RdRP* sequence analysis of SARS-CoV-2

We analyzed 19 SARS-CoV-2 sequences (Specimen 1, 2, 3, 5, 6, 7, 12, 13, 14, 15, 16, 17, 18, 19, 20, 21, 22, 23 and 25) collected from the first to third waves of COVID-19 outbreak in Hong Kong, and they were identical to SARS-CoV-2 Wuhan-Hu-1. We further analyzed 22,173 GISAID genomes contributed by 86 countries. SNVs were present in 961 sequences (4.33%), with majority (947/961, 98.54%) possessing a single SNV, 13 (1.35%) possessing 2 SNVs, and 1 (0.10%) harbouring 3 SNVs. These nucleotide variants comprised 3.00–4.87% of sequences from each month (Jan–May 2020), and 3.77–34.64% from 5 geographical regions (Table 4).

**Table 4:** Number of GISAID sequences by time, geographical regions and SNV status

Time	SNV	Africa	America	Asia/Middle East	Europe	Oceania	Subtotal	Total
Dec 2019	Absent	0	0	17	0	0	17	17
	Present	0	0	0	0	0	0	
Jan 2020	Absent	0	18	318	24	9	369	384
	Present	0	0	15	0	0	15 (3.91%)	
Feb 2020	Absent	1	108	418	122	17	666	688
	Present	0	2	18	1	1	22 (3.20%)	
Mar 2020	Absent	72	319	660	10,816	1006	12,873	13,435
	Present	22	63	21	407	49	562 (4.18%)	
Apr 2020	Absent	27	1,803	455	4233	219	6,737	7082
	Present	30	90	25	194	6	345 (4.87%)	
May 2020	Absent	0	118	87	330	15	550	567
	Present	1	2	6	6	2	17 (3.00%)	
Subtotal	Absent	100	2366	1955	15,525	1266	21,212	22,173
	Present	53	157	85	608	58	961	
Total		153	2523	2,040	16,133	1324	Global	
% with SNV		34.64	6.22	4.17	3.77	4.38	4.33	

**Fig. 2:**
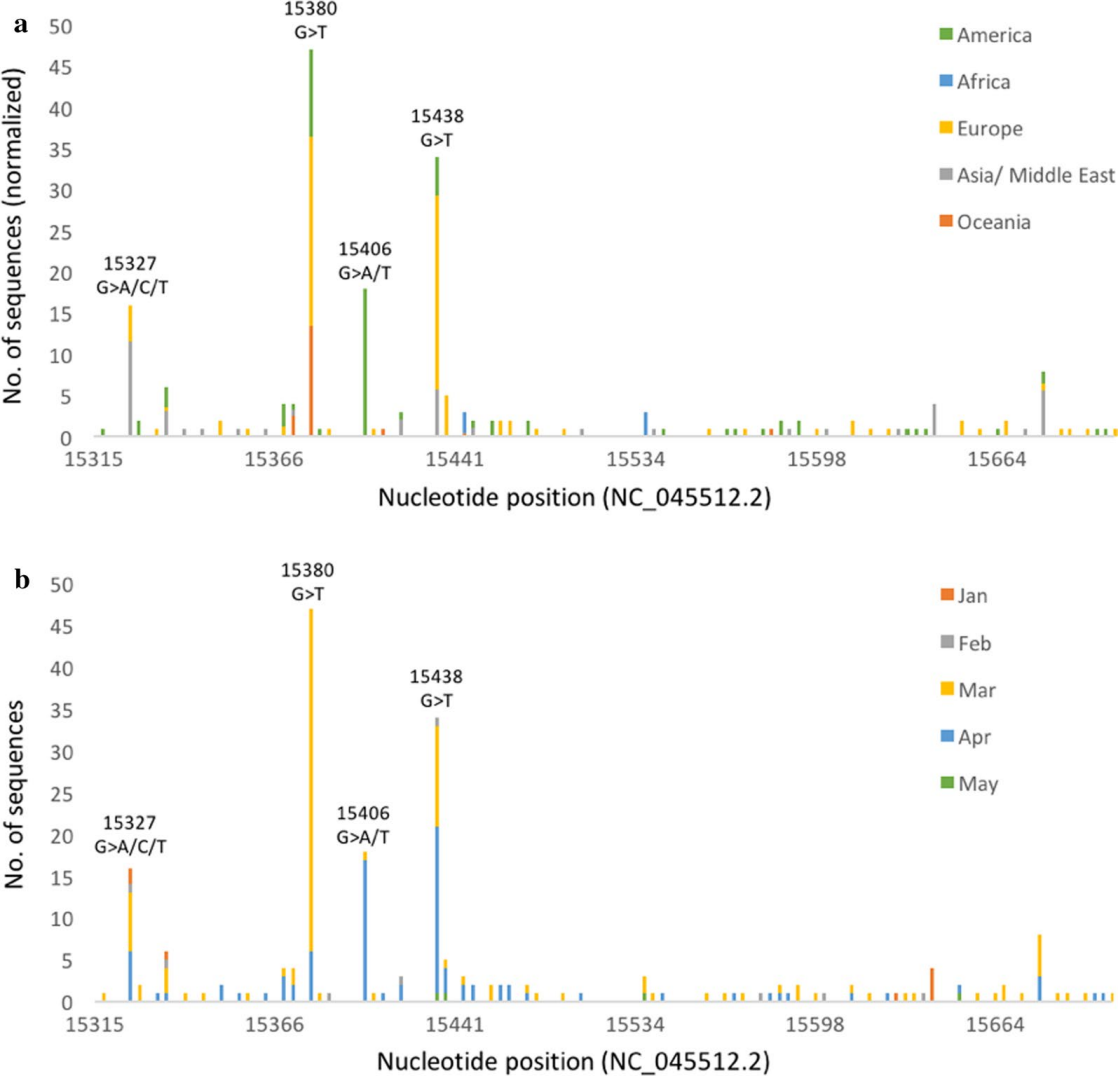
Missense mutations in partial *RdRP* gene of SARS-CoV-2 from Dec 2019 to May 2020. The number of sequences contributed by different **a** geographical regions (normalized) and **b** months are represented by different colours. The top 4 nucleotide positions with SNVs are labelled

A total of 125 SNV types involved 114 bases of *RdRP* gene (NC_045512.2:15315-15696), with more than half being missense mutations (71/125, 56.8%). For synonymous mutations, 15324C > T was the most common and present in 553 genomes. The frequencies of missense mutations were the highest at bases 15327 (n = 16), 15380 (n = 47), 15406 (n = 18) and 15438 (n = 34) with different geographical patterns (15327: Asia/ Middle East > Europe; 15380: Europe > Oceania > America; 15406: America > > Europe; 15438: Europe > Asia/ Middle East > America), and majority of these sequences were collected in March and April, 2020 (Fig. 2).

Table 5 lists the missense mutations and corresponding amino acid changes, using SARS-CoV-2 RdRP protein sequence YP_009725307.1 as reference. The 71 missense mutations affected rear part of RdRP (amino acid 627–752), with 9 SNVs found on 4 conserved domains, leading to amino acid changes at polymerase motif A (P627S), zinc binding site (H642N), non-structural protein 8 (nsp8) interaction site (M666I) and polymerase motif B (G683V, D684G, A699S, V700I, V700A and N705D). The frequency of M666I (15438G > T) was the highest (n = 34).

**Table 5:** Summary of missense mutations on partial ***RdRP*** gene of SARS-CoV-2 and corresponding amino acid changes. Nucleotide and amino acid positions correspond to reference sequences NC_045512.2 and YP_009725307.1, respectively

SNVs	Amino acid changes	No. of sequences	Remarks
15319C > T	P627S	1	^§^End of conserved polymerase motif A (17/17), containing the classic divalent-cation-binding residue D618, related to RdRP fidelity
15327G > A	M629I	2	
15327G > C	M629I	1	
15327G > T	M629I	13	
15328C > T	L630F	2	
15338T > C	M633T	1	
15346C > A	L636I	2	
15346C > T	L636F	4	
15349G > C	V637L	1	
15352C > T	L638F	1	
15356C > T	A639V	2	
15358C > A	R640S	1	
15359G > A	R640H	1	
15364C > A	H642N	1	^§^Zn binding site (6/8)
15368C > T	T643I	4	
15371C > T	T644M	4	
15380G > T	S647I	47	
15384G > T	L648F	1	
15392G > A	R651H	1	
15406G > A	A656T	17	
15406G > T	A656S	1	
15407C > T	A656V	1	
15412G > T	E658*	1	
15418G > T	A660S	3	
15438G > T	M666I	34	^§^End of nsp8 interaction site (52/52)
15439G > A	V667I	5	
15444G > T	M668I	3	
15448G > A	G670S	1	
15448G > T	G670C	1	
15451G > A	G671S	2	
15452G > T	G671V	2	
15460T > C	Y674H	2	
15463G > T	V675F	2	
15472G > T	G678C	1	
15488G > T	G683V	1	^§^Conserved polymerase motif B (4/30), related to RdRP fidelity
15491A > G	D684G	1	^§^Conserved polymerase motif B (5/30), related to RdRP fidelity
15535G > T	A699S	3	^§^Conserved polymerase motif B (20/30), related to RdRP fidelity
15538G > A	V700I	1	^§^Conserved polymerase motif B (21/30), related to RdRP fidelity
15539T > C	V700A	1	^§^Conserved polymerase motif B (21/30), related to RdRP fidelity
15553A > G	N705D	1	^§^Conserved polymerase motif B (26/30), related to RdRP fidelity
15571G > T	D711Y	1	
15574G > A	G712S	1	
15575G > T	G712V	1	
15586G > T	A716S	1	
15589G > T	D717Y	1	
15593A > G	K718R	2	
15594G > T	K718N	1	
15596A > G	Y719C	2	
15598G > T	V720F	1	
15602G > A	R721H	1	
15613C > T	H725Y	2	
15619C > T	L727F	1	
15627G > T	E729D	1	
15636T > A	Y732*	1	
15638G > A	R733K	1	
15640A > T	N734Y	1	
15641A > C	N734T	1	
15647A > G	D736G	4	
15652G > T	D738Y	2	
15656C > T	T739I	1	
15664G > A	V742M	1	
15665T > C	V742A	1	
15665T > G	V742G	1	
15668A > G	N743S	1	
15672G > T	E744D	8	
15682T > A	Y748N	1	
15683A > T	Y748F	1	
15685T > A	L749M	1	
15688C > T	R750C	1	
15689G > A	R750H	1	
15696T > G	H752Q	1	

^§^Depicted from NCBI Conserved Domain database [14]

## Discussion

We successfully characterized coronaviruses directly from majority of clinical specimens. For SARS-CoV-2, full-length *RdRP* sequences could be retrieved from specimens with Ct values of 31.68 (N gene) or less, suggesting that this method may be best used right after symptom onset when viral load is at its maximum [15]. Our data showed that highly accurate consensus sequences could be built from error-prone Nanopore reads if coverage depth was sufficient (> 30×). Considering the reference sequence of an unknown coronavirus is not readily available, we repeated consensus building for selected specimens without SARS-CoV-2 reference genome, and the consensus accuracy was not compromised.

From our experience, the universal primers used in this study amplified human and commensal sequences occasionally. As the non-specific band(s) was very close to the target, gel purification is required to obtain clean Sanger chromatograms. In this regard, Nanopore sequencing facilitates a simpler workflow as sequencing reads can be analyzed independently without gel purification. It may therefore provide better resolution for mixed coronavirus infection, which comprised about 4.3% of SARS-CoV-2-positive respiratory specimens from symptomatic patients [16]. Nanopore sequencing is also a faster option as the time from amplicons to sequence data is about half of the Sanger’s method. Compared with direct metagenomic sequencing, our method involved target enrichment by PCR and less complicated data processing, and consensus sequences were typically built in minutes. Using Flongle flow cells, reagent cost may be as low as 12 USD per sample for a 24-plex run [17], which is comparable to Sanger sequencing.

In general, the proportion of genomes possessing SNVs by geographical area (America, Asia/ Middle East, Europe and Oceania) and by month of collection (Jan-May 2020) were similar, ranging from 3.00 to 6.22% (Table 4) with the exception of Africa (34.64%). As 153 genomes were retrieved from Africa which was at least 8 times lower than other areas, this relatively high proportion of genomes with SNVs may require confirmation by more representative sampling.

**Fig. 3:**
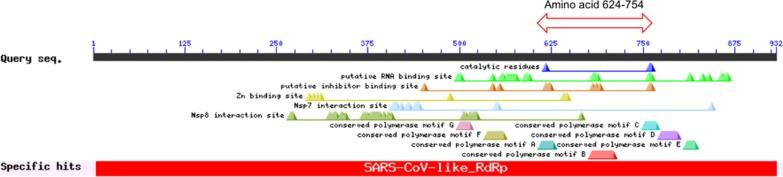
Second PCR amplicon flanking the codons for amino acid 624–754 of SARS-CoV-2 RNA-dependent RNA polymerase (YP_009725307.1). Related conserved domains are as shown (the image was adopted from NCBI Conserved Domains database [15])

The partial *RdRP* gene we targeted encompasses parts of conserved domains which are important to polymerase functionality (Fig. 3). Our data displayed the diversity of SNVs involving 114 bases (28.93%) in a short segment of 394 bp, and missense mutations generally occurred at low frequencies (ranged from 1 to 47 genomes) compared to 15324C > T synonymous mutation (n = 553). Among the missense mutations found on conserved domains, the frequency of 15438G > T was the highest (n = 34) which changes the last residue of cofactor nsp8 interaction site from methionine to isoleucine (M666I) and was predominantly found in Europe. As mutation is a two-edged sword, the effect of these missense mutations on the pathogenicity of SARS-CoV-2 awaits further investigation, and added knowledge in this area is important for development of antiviral drugs, vaccines and diagnostic assays.

This study had several limitations. First, the variety of HCoVs might not be sufficient for thorough evaluation of a ‘pan-coronavirus’ assay, and further studies with more comprehensive sample collection is warranted. As a portion of MinION flow cells possessed suboptimal number of active pores, the sequencing time of some specimens might be overestimated. As Nanopore consensus sequences were built by majority rule, minority SNVs present in the specimens might not be detected. In addition, as GISAID EpiCoV™ database is expanding continuously, there may be changes in geographical and temporal SNV patterns after accumulation of more SARS-CoV-2 genome data.

## Conclusion

We developed and evaluated a method for direct characterization of coronaviruses from respiratory specimens, based on pan-coronavirus amplification and sequencing of partial *RdRP* gene. It provides a viable option for first-line etiologic investigation of suspected infection by unknown coronavirus, which may lead to more timely follow-up actions. The SNV data shed light on global distribution and frequencies of missense mutations in partial *RdRP* gene of SARS-CoV-2, providing valuable information for surveillance of this important antiviral drug and diagnostic target.

## Data Availability

The datasets used and/ or analyzed during the current study are available from the corresponding author on reasonable request.

## Abbreviations

COVID-19: Coronavirus disease 2019
Ct: Threshold cycle
GISAID: Global Initiative on Sharing All Influenza Data
HCoV: Human coronavirus
IGV: Integrative Genomics Viewer
MERS: Middle East respiratory syndrome
N: Unknown base
N/A: Not available
ND: Not done
NPA: Nasopharyngeal aspirate
NPS: Nasopharyngeal swab
NS: Nasal swab
pOS: Posterior oropharyngeal saliva
RdRP: RNA-dependent RNA polymerase
REC: Research Ethics Committee
RP2plus: The BioFire® FilmArray® Respiratory 2 plus Panel
rRT-PCR: Real-time reverse transcription polymerase chain reaction
SARS-CoV-2: Severe acute respiratory syndrome coronavirus 2
seq.: Sequence
SNV: Single nucleotide variant
SP: Sputum
TS: Throat swab
VC: Virus culture
